# Supplementary material for: Land-Cover Classification Using MaxEnt: Can We Trust in Model Quality Metrics for Estimating Classification Accuracy?
Source: Entropy (Basel). 2020 Mar 17;22(3):342. doi: 10.3390/e22030342 (PMC7516803; doi:10.3390/e22030342)
Supplement: Supplementary file 1 [file entropy-22-00342-s001.zip › entropy-678448-supplementary/entropy-678448-supplement.docx]

**Supplementary Materials**

**Table 1.** Results of Shapiro-Wilk normality test for the distribution of all variables used for correlation analysis. Numbers in table are p-values of normality test. Only p-values >0.05 were considered normally distributed, which are highlighted in bold.

**.**

**
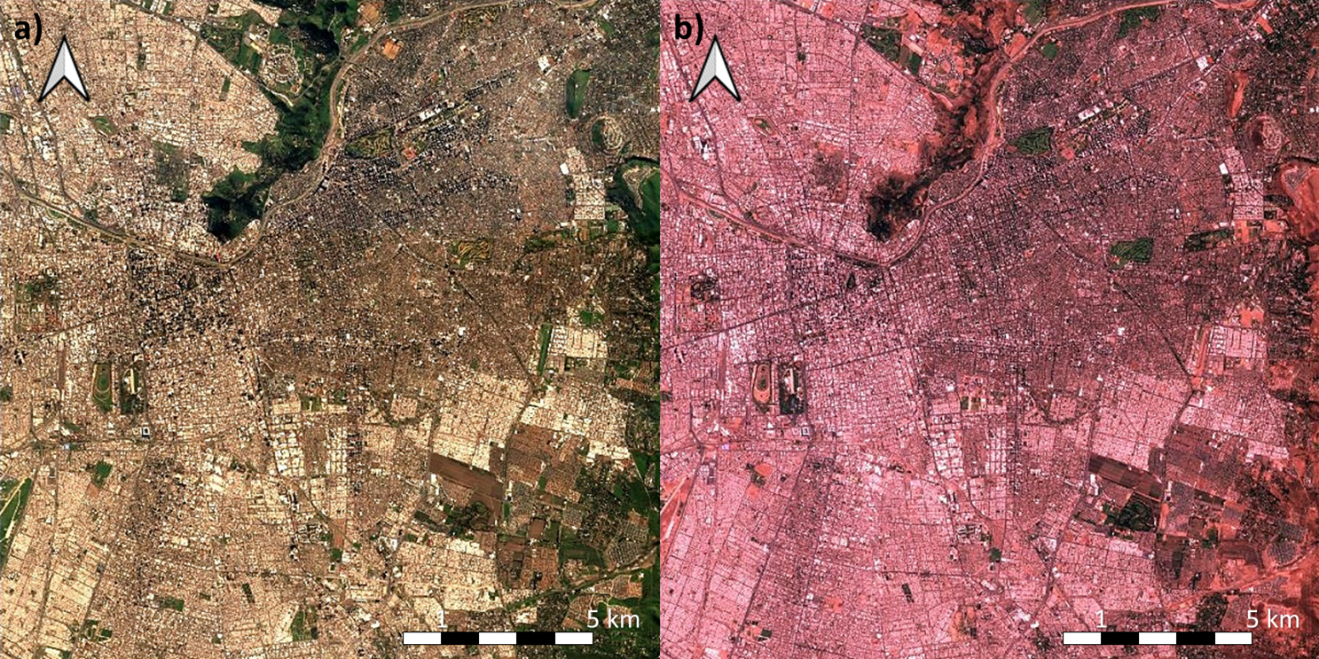
**

**Figure 1.** Composite Sentinel 2 satellite images of the 16 x 16 km quadrant used for testing the accuracy of classification results. Figures show winter phenological conditions in a) and summer phenological conditions in b).


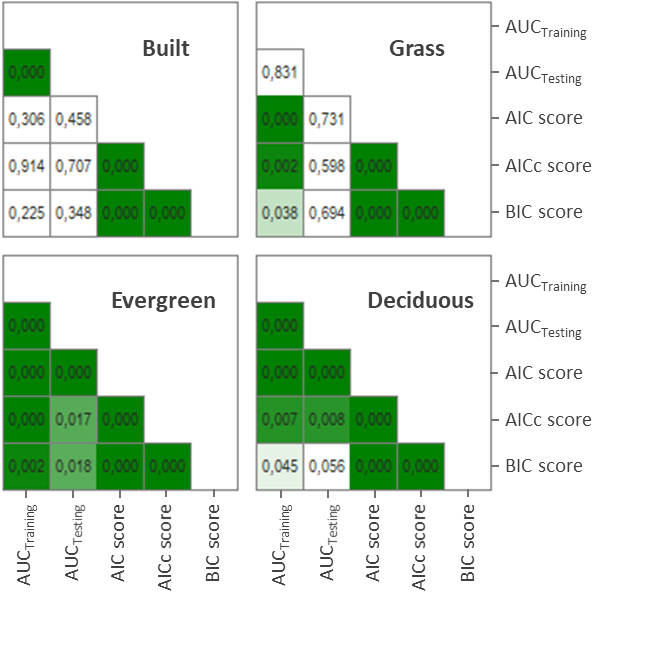
.

**Figure 2.** P-values for the Spearman correlation between the five MaxEnt model’s quality metrics for the four analyzed land-covers. Squares are colored based on p-values from white (no significant at p>0.05) to dark green (highly significant at p<0.001).


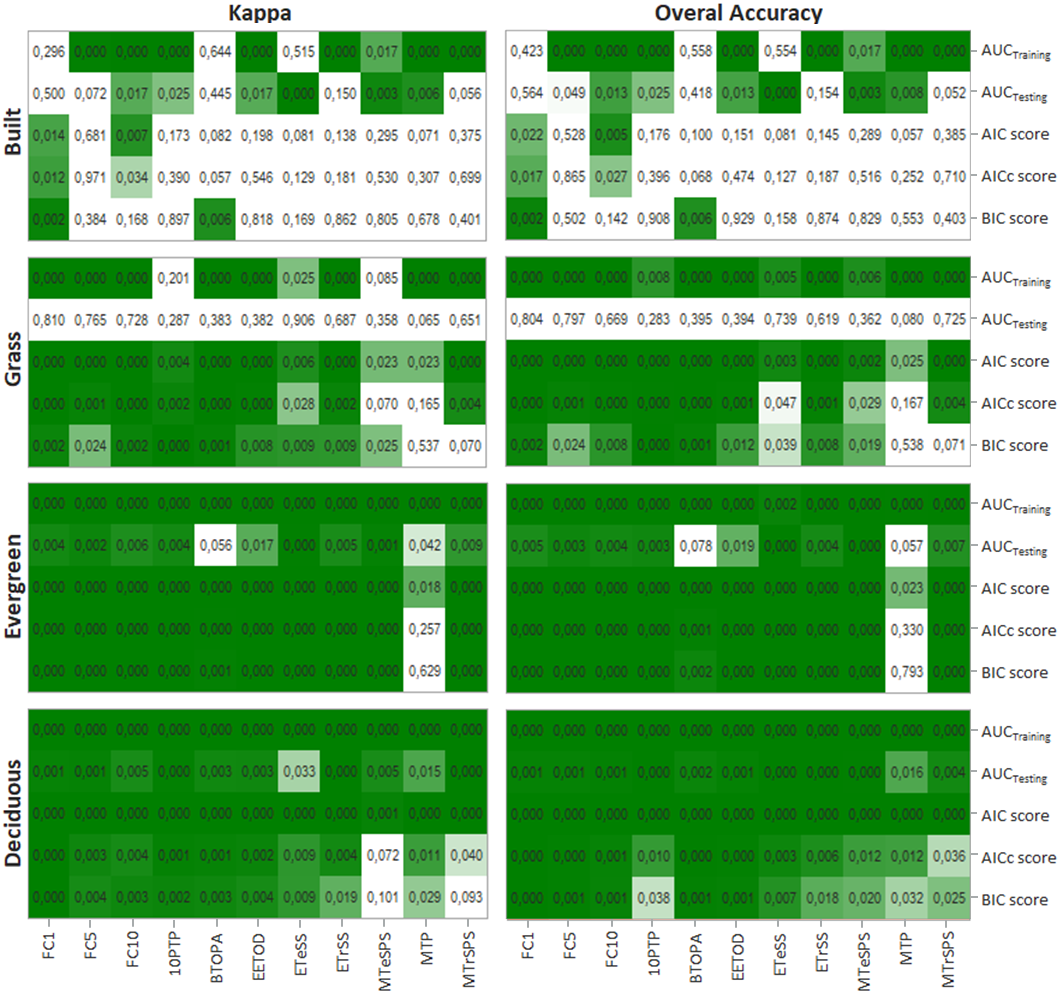


**Figure 3.** P-values for the Spearman correlation between the five MaxEnt model’s quality metrics and the two classification accuracy metrics for the eleven thresholds used for building the binary maps and the four analyzed land-covers. Squares are colored based on p-values from white (no significant at p>0.05) to dark green (highly significant at p<0.001).
